# Phase I/II Trial of Perioperative Avelumab in Combination With Chemoradiation in the Treatment of Stage II/III Resectable Esophageal and Gastroesophageal Junction Cancer

**DOI:** 10.1002/jso.28070

**Published:** 2025-01-05

**Authors:** Nataliya V. Uboha, Mustafa M. Basree, Jens C. Eickhoff, Dustin A. Deming, Kristina Matkowskyj, James Maloney, Daniel McCarthy, Malcolm DeCamp, Noelle LoConte, Philip B. Emmerich, Sean Kraus, Monica A. Patel, Jeremy D. Kratz, Sam J. Lubner, Newton Hurst, Michael F. Bassetti

**Affiliations:** ^1^ Division of Hematology, Medical Oncology and Palliative Care, Department of Medicine University of Wisconsin School of Medicine and Public Health Madison Wisconsin USA; ^2^ Carbone Cancer Center Madison Wisconsin USA; ^3^ Department of Human Oncology University of Wisconsin School of Medicine and Public Health Madison Wisconsin USA; ^4^ Department of Biostatistics and Medical Informatics University of Wisconsin School of Medicine and Public Health Madison Wisconsin USA; ^5^ William S. Middleton Memorial Veterans Hospital Madison Wisconsin USA; ^6^ Department of Pathology and Laboratory Medicine University of Wisconsin School of Medicine and Public Health Madison Wisconsin USA; ^7^ Division of Cardiothoracic Surgery, Department of Surgery University of Wisconsin School of Medicine and Public Health Madison Wisconsin USA; ^8^ Center for Human Genomics and Precision Medicine University of Wisconsin Madison Wisconsin USA

**Keywords:** chemoradiation, esophageal cancer, gastroesophageal adenocarcinoma, immunotherapy

## Abstract

**Background and Objectives:**

Standard treatment of patients with stage II/III esophageal or gastroesophageal junction (E/GEJ) cancer involves neoadjuvant chemoradiation (nCRT), resection, and immunotherapy. Our trial evaluated the addition of perioperative avelumab to standard treatments.

**Methods:**

Patients with resectable E/GEJ cancers received avelumab with nCRT and adjuvant avelumab after resection. Primary endpoints for phase I and II portions were safety and pathologic complete response (pCR) rate, respectively. Secondary endpoints included recurrence‐free survival (RFS), surgical complication prevalence, and R0 resection rate.

**Results:**

Twenty‐two patients enrolled in the study. Median follow‐up during data cutoff was 23.9 months. There were no dose‐limiting toxicities during the run‐in phase. Nineteen patients (86.4%) underwent resection with R0 resection rate of 78.9% and with pCR rate of 26%. Most common treatment‐related adverse events (TRAE) were cytopenias from chemoradiation. Aside from one grade ≥ 3 avelumab‐related hypersensitivity, no grade ≥ 3 avelumab TRAEs were seen. Median RFS was not reached, and 1‐year RFS and overall survival were 71% and 81%, respectively. The study was terminated before full planned accrual due to standard practice change based on the CheckMate 577 trial.

**Conclusions:**

The addition of perioperative avelumab to nCRT was tolerable and demonstrated promising outcomes.

## 1 Introduction

Esophageal and gastroesophageal junction (E/GEJ) cancers are aggressive malignancies that contribute to cancer‐related mortality globally. There are roughly 22 300 cases of E/GEJ diagnosed annually in the United States, with 16 130 deaths from E/GEJ cancer annually [1]. The majority of patients present with at least stage II disease, thus requiring multimodality therapy. Despite aggressive treatments, about 50% of patients ultimately develop recurrent disease [2, 3], with both local and distant progression, the highest risk being in those without tumor downstaging after neoadjuvant chemoradiation (nCRT) [4].

Effective management requires a comprehensive and multidisciplinary approach, particularly for early‐stage upper gastrointestinal (UGI) cancers. Standard of care for managing patients with E/GEJ involves nCRT followed by surgical resection, as established by the CROSS trial [5]. This approach provides a 13% absolute benefit in long‐term survival over 10 years (38% vs. 25% with surgery alone, *p* = 0.004) [2]. While nCRT improves R0 resection rates and reduces lymph node involvement, observed pathologic complete response (pCR) is low at 29%. While an imperfect surrogate, pCR rate at time of resection is important as it correlates with survival [4, 6, 7]. Patients without pCR, especially those with ypN+ at resection, face a higher risk of recurrence [4, 8].

Immuno‐oncology (IO) agents, such as immune checkpoint inhibitors, have emerged as powerful tools in the treatment of patients with E/GEJ cancers. Both nivolumab and pembrolizumab (antibodies against programmed cell death protein 1) are now approved in the metastatic setting in combination with chemotherapy based on the results of the phase III trials that demonstrated survival advantage with the addition of these agents [9, 10]. In addition, the combination of ipilimumab and nivolumab has established activity for esophageal squamous cell carcinomas (ESCC) as well [11]. In the early‐stage setting, 12 months of adjuvant nivolumab is now recommended for patients with residual pathologic disease after nCRT, based on the results of a global phase III CheckMate 577 trial. This trial demonstrated improvement in median recurrence‐free survival (RFS) with adjuvant nivolumab use when compared to placebo (22.4 vs. 11.0 months, hazard ratio 0.69; *p* < 0.001) in a biomarker nonselect patient population [3].

Thus, given the established activity of immune checkpoint inhibitors across disease stages, incorporating them early during the treatment, including with chemoradiation, may increase the cure rate for patients with E/GEJ cancers. The goal of this study was to evaluate the safety and preliminary efficacy of perioperative avelumab, an anti‐programmed cell death ligand protein 1 antibody, when combined with neoadjuvant chemoradiation before esophagectomy in the treatment of E/GEJ cancers. This study was launched before the approval of nivolumab in the early‐stage setting, and thus its design relied on standard practices during the time of its launch.

## 2 Materials and Methods

### 2.1 Study Design

This was a single arm, two‐part, open‐label, phase I/II investigator‐initiated trial conducted at the University of Wisconsin Carbone Cancer Center (NCT03490292). Part 1 was a run‐in phase that enrolled six patients with a primary endpoint of safety and tolerability of avelumab in combination with neoadjuvant chemoradiation. The second part, the expansion phase II cohort of the trial, planned to enroll 18 additional patients to evaluate the activity of the proposed treatment and to obtain further safety information. The primary endpoint of the phase II portion was pathologic complete response rate (pCR) rate. Secondary endpoints included RFS, rate of surgical complications, and rate of negative margin (R0) resection. Recurrences were monitored with restaging scans and were confirmed with biopsies when clinically indicated. CT scans were obtained every 4 months during the first 12 months after all treatment completion. After the final surveillance visit at 12 months postadjuvant therapy completion, patients were followed per discretion of their treating provider. Survival and disease status data will be collected over a time period of up to 3 years from medical records and phone interviews.

### 2.2 Patient Selection

Patients with histologically confirmed, potentially curable E/GEJ squamous‐cell carcinoma, adenocarcinoma, or large‐cell undifferentiated carcinoma with clinical stage T1N1 or T2‐T3N0‐2, with no evidence of metastatic spread, and who were candidates for neoadjuvant chemoradation (nCRT) treatment and resection at the time of consent signing were eligible to participate. Staging procedures included positron emission tomography/computed tomography (PET/CT) and endoscopic ultrasound. Patients were required to have good organ function with no contraindications for immunotherapy or RT. Patients with cervical esophageal carcinoma were excluded.

The study was conducted in accordance with all applicable regulatory requirements, and the protocol was approved by the University of Wisconsin‐Madison Health Sciences Institutional Review Board. All patients provided written informed consent before study enrollment. The study complied with international standards of Good Clinical Practice and the Declaration of Helsinki.

### 2.3 Treatments

Patients were treated with nCRT with weekly paclitaxel (50 mg/m^2^) and carboplatin (AUC 2). Chemotherapy started on Day 1 of RT and was given every 7 days. A total of 5 weekly chemotherapy treatments were planned during the study. Radiotherapy was delivered to a total dose of 41.4 Gray (Gy) in 23 fractions. Both 3‐D conformal radiotherapy (3D‐CRT) and intensity‐modulated radiotherapy (IMRT) were allowed. Avelumab (10 mg/kg) was administered intravenously every 2 weeks. This dose was selected based on the ongoing studies at that time with avelumab enrolling patients with gastroesophageal cancers [12]. Three doses of avelumab (starting with the last weekly chemotherapy administration) were administered before surgery. If the last dose of chemotherapy was omitted due to cytopenias, avelumab was still administered as originally planned. Surgical resection was performed 8‐10 weeks after the completion of chemoradiation. The type of surgical resection was left to the surgeon's discretion. Postoperatively, six doses of avelumab (10 mg/kg administered every 2 weeks) were administered in adjuvant setting. All patients received standard‐of‐care supportive medications as per institutional standards.

### 2.4 Safety Evaluation

The first six patients were evaluated for dose‐limiting toxicities (DLTs) of avelumab in combination with chemoradiation. DLT evaluation lasted until the first postoperative clinic visit at about 2–4 weeks after resection. DLT evaluation was performed by a treating medical oncologist in discussion with a multidisciplinary team when appropriate. For the purposes of this trial, DLTs were defined as an adverse event (AE) that occurred after avelumab administration during the perioperative period that was clinically significant and/or unacceptable, such as interfering with standard of care therapy, including safe surgical resection, and judged to be related to the avelumab treatment. With respect to surgical complications, particular focus was given to the events in the immediate postoperative period. We also evaluated potential surgery delays after utilization of avelumab in combination with neoadjuvant chemoradiation.

### 2.5 Pathological Evaluation

Tissue sections of resected specimens were evaluated to assess pathologic response. The study defined pCR as the absence of any viable tumor at microscopic examination of the primary tumor and lymph nodes sampled postoperatively following neoadjuvant therapy. In patients without pCR, measurement of residual disease was performed. Tumor regression grade (TRG) was used to assess treatment effects and classified into four categories: score 0, no viable cancer cells (pCR); score 1, single cells or rare small groups of cancer cells (near complete response); score 2, residual cancer with evident tumor regression, but more than single cells or rare small groups of cancer cells (partial response); score 3, extensive residual cancer with no evident tumor regression (poor or no response).

### 2.6 Sample Size and Statistical Considerations

A total of six subjects were enrolled during the run‐in phase of the trial. A sample size of six was sufficient to estimate the true DLT rate of the proposed avelumab/chemoradiation therapy with adequate accuracy. Specifically, the true DLT rate was estimated with a standard error of 20%. The proposed treatment combination was considered as safe if DLTs were observed in at most one patient. The expansion portion of the trial planned to enroll 18 patients. Both safety cohort and expansion cohort patients, a planned total of 24, were evaluated in efficacy assessment. The null hypothesis that the pCR rate is at most 20% was tested against the alternative hypothesis that pCR was > 20%. With a sample size of 24 patients, an anticipated pCR rate of 40% could be detected with 80% power at one‐sided 0.1 significance level. Anticipated pCR rate of 40% was similar to the design of EA2174 trial, which is a phase 2/3 that hypothesized that nivolumab addition to nCRT can increase pCR rate by 15% [13]. In addition, CALGB 80803 study demonstrated that pCR rate in the experimental group with the best outcomes was 40.3% [14]. Descriptive statistical analysis was used to evaluate RFS, rate of surgical complications, and rate of negative margin (R0) resection, and pathological outcomes, while relying on historical references for comparisons.

Survival analysis was performed using Kaplan Meier estimates.

### 2.7 Correlative Studies

Tissue and blood samples were collected throughout treatment for correlative studies. Blood samples were collected at the time of avelumab treatment initiation, before the third dose of avelumab during preoperative period, before the first and last adjuvant avelumab dose, and at the last planned surveillance visit ~ 12 months after adjuvant therapy completion, These samples are currently stored for future correlative studies that are being planned. Immunohistochemistry (IHC) studies were performed on formalin‐fixed/paraffin‐embedded (FFPE) tumor tissue. Collected tissue included pretreatment biopsies, as well as posttreatment resection specimens (when available). Programmed death‐ligand 1 (PD‐L1) combined positive score (CPS) was performed on all available tissue as per standard of care, utilizing DAKO 22C3 clone. Versican (VCAN) proteolysis was examined as a potential predictive and prognostic immune biomarker [15, 16, 17]. IHC was performed for the total (intact) VCAN and a proteolyzed VCAN product called versikine. Biomarker quantification was performed in quartiles (0, 1 + , 2 + , or 3 + ) with lower number denoting weaker staining. VCAN proteolytic states for each sample were determined as either VCAN proteolysis predominant (both VCAN < 2+ and versikine > 2 + ) or VCAN proteolysis weak (either VCAN > 2 + or versikine < 2 + ). CD8 + T‐cells were quantified per high powered field (40x) in both epithelial and stromal components of the tumor bed and averaged across five independent fields of view.

## 3 Results

### 3.1 Patient Characteristics

Twenty‐two patients were enrolled in this study between August 6, 2018 and February 2, 2022, with a median follow‐up of 23.9 months (range, 1–53 months) at time of data cutoff of July 13, 2023. Full patient characteristics and demographics are listed in Table 1. Study cohort had a median age of 64 years (range, 42–76 years), was predominately male (91%), Caucasian (100%), with adenocarcinoma histology (86%), and with 64% having GEJ tumors. Most patients had cT3 (95%) and cN+ (63%) disease at the time of enrollment. Of note, the study enrollment was terminated before full planned accrual due to change in standard practice based on the CheckMate 577 trial.

**Table 1:** Baseline patient characteristics (*n* = 22).

Variables of Interest	*n* (%)
Age, Median (Range), years	64 (42–76)
Gender, *n* (%)	
Male	20 (91%)
Female	2 (9%)
Race, *n* (%)	
Caucasian	22 (100%)
Other	0 (0%)
ECOG PS	
0	12 (55%)
1	10 (45%)
2	0 (0%)
Tumor Location	
Esophageal	8 (36%)
GEJ	14 (64%)
Histology	
Squamous	3 (14%)
Adenocarcinoma	19 (86%)
Clinical Staging	
cT2	1 (5%)
cT3	18 (95%)
cN0	7 (37%)
cN+	12 (63%)

Abbreviations: ECOG PS, Eastern Cooperative Oncology Group Performance Status; GEJ, gastroesophageal junction.

### 3.2 Safety Evaluation

There were no DLTs during the run‐in phase. There were no unexpected surgical complications. All subjects experienced at least one TRAE, the majority of which were reversible and not attributable to avelumab (Table 3). Most common grade ≥ 3 TRAEs were lymphopenia (95.5%), leukopenia (59.1%), and neutropenia (13.6%). There were no definite grade ≥ 3 immune‐related AEs; of note, one patient developed grade 3 hypersensitivity reaction and was taken off study after one dose of avelumab in the preoperative period. Most grade 1 and 2 toxicities were directly related to nCRT, with bone marrow suppression related to chemotherapy and esophagitis related to RT being the most common (Table 2). Three patients developed grade 1–2 avelumab‐related hypothyroidism. Three patients experienced grade 2 hypersensitivity reaction to avelumab, but this did not result in drug discontinuation.

**Table 2:** Grade ≥ 3 treatment‐related adverse events (*n* = 22).

Grade ≥ 3 TRAE	*n* (%)
Lymphopenia	21 (95%)
White blood cell decrease	13 (59%)
Neutropenia	6 (27%)
Diarrhea	3 (14%)
Acute Kidney Injury	1 (5%)
Hypotension	1 (5%)
Diarrhea	1 (5%)
Dehydration	1 (5%)
Infusion reaction	1 (5%)
Nausea	1 (5%)

Abbreviation: TRAE, treatment‐related adverse events.

### 3.3 Efficacy Evaluation

Out of 22 patients, 19 completed neoadjuvant treatment and underwent resection as part of the study and 16 completed adjuvant immunotherapy as part of the study. Of the 3 patients who did not undergo resection on the study, one was taken off protocol due to a grade 3 hypersensitivity reaction related to avelumab. This patient was subsequently able to complete his treatment, which included resection and adjuvant nivolumab as per standard of care. Another patient withdrew consent from the study before resection per personal preference. The third patient was removed from study due to protocol non‐adherence. The latter two patients were able to undergo resection as per standard of care. Among the evaluable 19 patients, there were no unexpected surgical complications, with similar rates of surgical complications to those observed in our institution previously (summarized in Table 3) In the postoperative period, one patient did not receive adjuvant avelumab due to positive margin at the time of resection. This patient was treated off protocol. Two additional patients did not receive adjuvant therapy due to postoperative complications (one patient developed necrosis of the large bowel requiring additional resection and one patient had anastomotic leak, thrombotic events, wound infection, and persistent pleural effusion complicating postoperative recovery).

**Table 3:** Surgical procedures and outcomes.

Type of surgery:	*N* = 19
Minimally Invasive Ivor‐Lewis esophagogastrectomy	13
Transhiatal esophagectomy	1
Three‐field (McKeown) esophagectomy	1
Open Ivor‐Lewis esophagectomy	4
Surgical complications*:	
No complications	11
< 30 days*	
Anastomotic leak	1
Atrial fibrillation	1
Acute thrombotic event	1
Wound infection	1
Bronchoesophageal fistula requiring stenting	
30–90 days	
Anastomotic stricture	2
Median time to adjuvant therapy initiation (days)	68

* Some of the complications occurred in the same patient.

**Figure 1:**
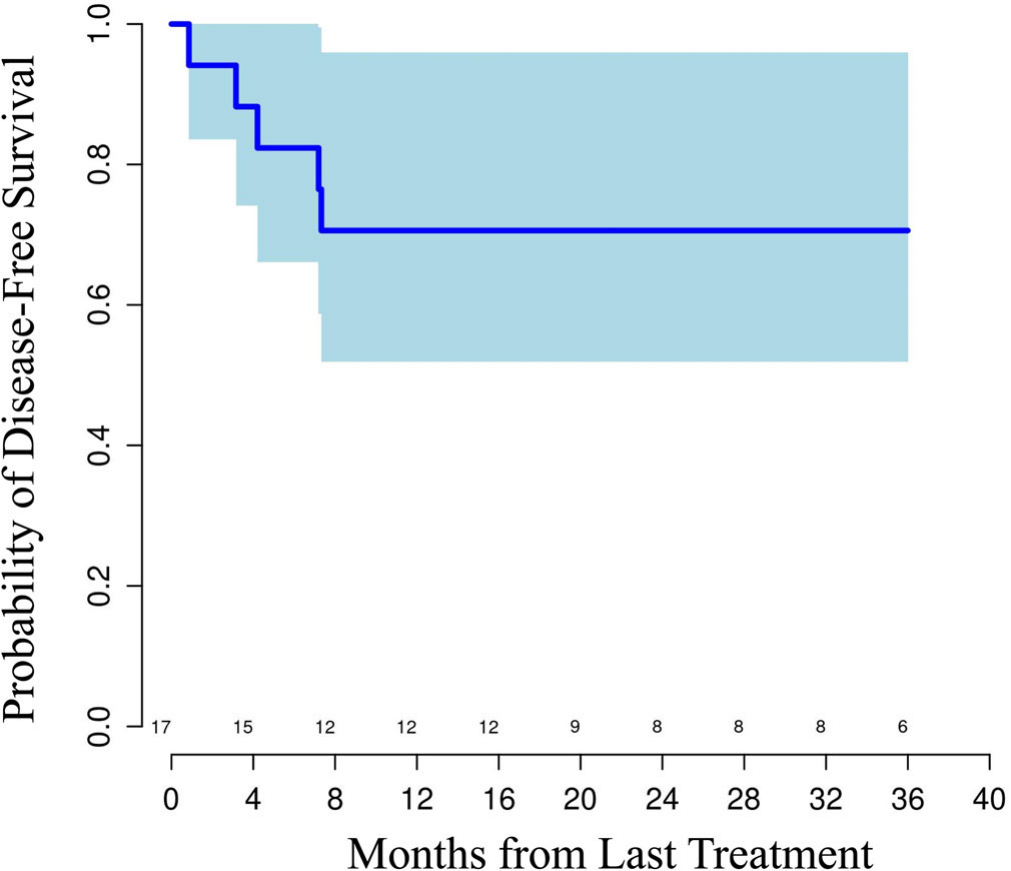
Kaplan‐Meier curve of recurrence‐free survival (solid line) with 95% confidence interval boundaries.

On pathologic evaluation the majority of patients had R0 resection (*n* = 15; 79%). Among the four patients with a margin positive (R1) resection, three had a positive radial resection margin and one had a positive proximal margin. The median number of lymph nodes removed was 19, with interquartile range of 18–24. Considerable tumor downstaging was observed in most patients (Table 4). Fourteen (74%) patients had node negative disease at resection. Tumor regression score of 0 or 1 (complete or near complete response, respectively) was noted in eight patients (42.1%); pCR rate was 26% (*n* = 5/19 patients; 3/16 E/GEJ adenocarcinoma (EAC) and 2/3 ESCC). Interestingly, a patient with positive proximal resection margin had microsatellite unstable tumor and had no evidence of treatment response on pathology. Median RFS was not reached at the time of data cutoff after a median follow‐up period of 23.9 months (range 1–53+ months). Estimated Kaplan‐Meier 1‐year RFS and overall survival (OS) were 71% and 81%, respectively (Figure 1). Five patients developed recurrent disease during the time of follow‐up. One of these patients had lung metastases, which were treated with locoregional therapies, and this patient remains free of disease as of data cut off.

**Table 4:** Pathological outcomes (*n* = 19 evaluable patients).

Pathologic outcomes		*n* (%)
Surgical staging	ypT0	5 (26%)
	ypT1	4 (21%)
	ypT2‐3 (%)	10 (53%)
	ypN0	14 (74%)
	ypN+	5 (26%)
Pathologic response score	0	5
	1	3
	2	11
Resection margins	R0	15 (79%)
	R1	4 (21%)^1^
Path complete response rate	All patients	5 (26%)
	Adenocarcinoma (*N* = 16)	3 (19%)
	Squamous (*N* = 3)	2 (67%)

Abbreviation: yp, pathologic staging after neoadjuvant therapy.

^1^ Three of the patients with R1 resection had positive radial resection margin, which is not considered a true surgical margin.

### 3.4 Correlative Studies

Available tumor sections were evaluated for versican and versikine expression, versican proteolysis status (VPP), PD‐L1 CPS, and CD8 + T‐cell infiltration (Supporting Information S1: Table S1). Limited tumor tissue was available at the time of resection. As such, correlative studies were performed on treatment‐naïve samples. A total of four patient's cancers were found to have the VPP phenotype. The complete pathologic response rate was 50% for this population and only one of these patients developed recurrent disease. A much larger sample size is required to formally evaluate VPP as a predictive biomarker. In terms of PD‐L1 CPS, all patients had tumors with very low PD‐L1 expression (every tumor had a PD‐L1 CPS score < 5 on pretreatment biopsy). This is a surprising finding given the previously reported status of PD‐L1 in the literature in these tumors [10, 18]. However, this marker was not particularly relevant to our study. CD8 + T cell infiltration demonstrated a wide range across a small number of samples, again limiting its interpretation [17, 19].

## 4 Discussion

Our study explored the incorporation of perioperative avelumab with nCRT for resectable E/GEJ cancers. Avelumab is an anti‐PD‐L1 antibody, and we did not expect that avelumab efficacy would necessarily differ from other immune checkpoint inhibitors utilized in the treatment of disease. Rather, this trial posed a question of timing. In our study, we assessed safety and preliminary efficacy of avelumab when it is incorporated earlier in the treatment paradigm for resectable E/GEJ cancers and in combination with nCRT. The results yielded encouraging outcomes, including a favorable safety profile, allowing for successful resection in most patients, and promising 1‐year RFS and OS rates. Observed TRAEs were largely due to nCRT and were expected with this treatment strategy. These TRAEs consisted primarily of reversible hematologic toxicities and did not result in unexpected complications or treatment delays.

The addition of perioperative avelumab to nCRT had a pCR rate of 26%, similar to findings in the CROSS trial (29%) and recently to that of pathologic analysis from the ECOG‐ACRIN EA2174 study [20]. As noted earlier, in prior studies pCR at time of resection was associated with better survival [4, 6, 7]. Patients who have residual disease at the time of resection, especially those with ypN + , are at a significant risk for recurrence [8]. The recurrences are predominately distant, outside the RT field [2, 9, 21]. Across studies, lymph node clearance has emerged as one of the most important factors associated with improved survival [22, 23]. In our study, 63% of patients had LN involvement on initiation staging and only 26% had positive LN at the time of resection.

Notably, this study protocol was developed before the incorporation of immune checkpoint inhibitors into the treatment paradigms for early‐stage disease. However, patient enrollment coincided with the reporting of CheckMate 577, which established adjuvant nivolumab as the standard of care for patients with resectable E/GEJ cancers (both adenocarcinomas and squamous cell cancers were allowed) who completed nCRT and had residual disease at time of resection [3]. Only patients who had residual pathologic disease after nCRT were eligible for participation and were randomized to 12 months of adjuvant nivolumab versus placebo. In our study design avelumab was administered adjuvantly for a planned duration of six cycles (3 months), mirroring perioperative treatment paradigms of gastric cancer hoping to deliver a total of 4‐6 months of perioperative systemic therapy [24]. Approval of nivolumab based on Checkmate 577 results resulted in early study termination, which may have impacted study results, especially when three out of 22 patients were not evaluable. Nonetheless, it remains an open question as to whether there is a potential superiority of perioperative versus adjuvant immunotherapy use in this patient population, especially when combined with radiation therapy. We hypothesize that perioperative IO may provide control against early disease dissemination, especially since chemotherapy used in nCRT is largely radiosensitizing and does not have a major impact outside the radiation field. Unfortunately, due to the study's small size, short follow‐up, and shorter duration of adjuvant avelumab, definitive conclusions regarding distant disease recurrence remain elusive. However, it is important to note that this strategy did not result in increased toxicities, an important consideration in a patient population treated with an operation associated with high morbidity that can limit tolerance of perioperative systemic therapies. Moreover, administration of adjuvant therapy was not delayed.

There are ongoing efforts to answer the question of benefit from IO addition to nCRT. This interest is fueled by growing preclinical and clinical data demonstrating synergism between radiation and immunotherapy agents, suggesting that combining these approaches may enhance antitumor activity and increase treatment efficacy [25, 26, 27]. Several studies for patients with early‐stage ESCC are investigating this question. An exploratory phase II is evaluating this synergism and omitting chemotherapy altogether in patients with resectable ESCC (NCT05176002). An earlier phase I study of 20 patients, PALACE‐1, laid the groundwork for immunotherapy combined with radiation therapy in those patients where perioperative pembrolizumab was concurrently administered along with nCRT per the CROSS regimen [28]. A follow‐up phase II study (PALACE‐2) is underway with a plan to enroll 143 patients (NCT04435197). NICE‐2 is an ongoing three‐arm phase II study evaluating IO + chemotherapy versus IO + nCRT versus nCRT alone in resectable ESCC (NCT05043688). There are many more IO‐chemo‐RT combination trials in ESCC ranging from phase I to III, nicely summarized in this review [29]. Hopefully, if the combination of nCRT and IO agents does prove to be superior to current standards in ESCC, future studies can focus of exploring organ preservation approaches and will allow patients to avoid morbidity and quality of life issues resulting from esophagectomy.

There are emerging data with this approach in E/GEJ adenocarcinomas as well. Addition of durvalumab to PET‐directed chemoradiation demonstrated acceptable safety and preliminary promising efficacy in advanced esophageal adenocarcinoma [30]. Neoadjuvant nivolumab and relatlimab (lymphocyte‐activation gene 3 (LAG‐3) antibody) in combination with chemoradiotherapy demonstrated promising efficacy in a phase 1B study [31]. Furthermore, MD Anderson Cancer Center has launched a phase I/II trial evaluating the efficacy of neoadjuvant‐modified FOLFOX (oxaliplatin and 5‐fluorouracil [5‐FU]) with concurrent and adjuvant atezolizumab with or without tirafolumab, an anti‐TIGIT agent in patients with resectable E/GEJ cancers (NCT03784326). Ongoing prospective phase II/III EA2174 study (NCT03604991) will be able to answer the question regarding perioperative use of IO in the treatment of esophageal and GEJ adenocarcinoma in a more definitive manner. Although interim analysis of this study demonstrated no improvement in pCR with the addition of IO therapy to nCRT, whether there are benefits on survival outcomes remain to be determined. Hopefully, the ongoing randomized study EA2174 evaluating nivolumab in a similar setting will provide a more definitive answer regarding the utility of this approach [20]. We are learning from gastric cancer trials that pCR, while a valuable marker of treatment response and tumor biology, does not necessarily translate into long‐term efficacy. KEYNOTE‐585, an international phase III trial that enrolled about 800 patients with untreated, locally advanced, resectable gastric or GEJ adenocarcinomas that were randomized 1:1 to perioperative chemotherapy with pembrolizumab or placebo, demonstrated improvement in pCR rate with pembrolizumab addition [32]. However, this pCR benefit did not translate into statistically significant (although numerically longer) RFS and OS benefit. It remains to be determined whether survival benefits are seen with improvement in pCR rates with the addition of durvalumab in a similarly designed MATTERHORN study [33].

While there are signs of early efficacy and strong preclinical data to support the combination of IO therapy and nCRT, it is important to note that recently presented ESOPEC data suggest that patients with resectable E/GEJ adenocarcinomas should be treated with FLOT chemotherapy as a preferable perioperative approach [34]. In the ESOPEC study, treatment with perioperative chemotherapy, rather than nCRT per CROSS, resulted in significantly improved OS in patients with resectable esophageal adenocarcinoma. Although this study did not incorporate adjuvant nivolumab in the control arm that received nCRT due to the timing of trial enrollment, it does suggest that our paradigm on how we approach these tumors should be updated, at least while we await final OS data from CheckMate 577. As such, it remains to be determined whether a combination of IO therapy and nCRT will be a relevant approach for resectable E/GEJ adenocarcinomas. Biomarkers remains an important area of research trying to delineate how best to select patients likely to benefit from IO incorporation into treatment paradigms. PD‐L1 CPS has an established role for selecting patients for IO therapy in the metastatic setting, which is most relevant to adenocarcinomas. Adjuvant nivolumab after nCRT and resection of E/GEJ cancer is approved regardless of PD‐L1 positivity. However, it should be noted that in a subgroup analysis in the CheckMate 577 trial, benefit was primarily seen in patient with PD‐L1 CPS ≥ 1 tumors [3]. In addition, PD‐L1 expression has been shown to be associated with greater depth of muscle invasion, presence of lymph node metastasis, and overall worse prognosis especially in patients treated with nCRT [10, 11]. Expression of PD‐L1 and other immune biomarkers can also change after radiation, further pointing towards potential synergism between radiation therapy and IO incorporation [35, 36]. These data, therefore, support exploring the role of IO agents, such as avelumab, in combination with nCRT to address systemic risk of disease spread and downstage primary tumor, and to reduce the radiation field, thereby lowering toxicity profile. This strategy could potentially be evaluated in patients who are not surgical candidates or in organ preservation studies in the future. Although limited biomarker studies were performed as part of our studies, with a small sample size, definitive conclusions cannot be made. Notably, PD‐L1 CPS was very low across all tumors at diagnosis, suggesting that it may not be a useful biomarker for IO use in locoregional disease if evaluated before treatment initiation. Notably, PD‐L1 expression has been shown to change after nCRT and thus may need to be evaluated at different timepoints during the treatment continuum [35]. Alternatively, with the study population having low PD‐L1 CPS, higher VCAN, and very low VPP status, all of which have been shown to predict for less response to immunotherapies in prior research, one may view the promising survival curve observed in the present study as a testament of this strategy to convert so called “cold” tumors to “hot” tumors when IO agents are combined with radiation therapy. Interestingly, one patient with microsatellite instability‐high (MSI‐H) tumor had minimal response to nCRT and rapid recurrence. While this was a surprising finding, this points to tumor heterogeneity in upper GI cancers and distinct response to IO agents in MSI‐H/mismatch repair deficient tumors of non‐colorectal primaries compared to MSI‐H colorectal cancers.

Our study has several limitations that warrant consideration. First, the inherent weaknesses in phase I/II studies with a relatively small sample size and single‐arm design coupled with early termination of the phase II expansion cohort limit the generalizability of our findings. Administering a maximum of six cycles (3 months) of adjuvant avelumab potentially negatively influenced our recurrence outcomes, considering the current standard is 12 months of adjuvant nivolumab per CheckMate 577 [3]. Enrollment of tumors of variable histology also limits interpretation as adenocarcinomas and squamous cell carcinomas have different biologic behavior and differential response to nCRT. Despite these limitations, our study possesses notable strengths. The incorporation of perioperative avelumab in combination with nCRT represents a novel approach that adds valuable insights to the evolving landscape of E/GEJ cancer management. Successful resections in most patients and promising 1‐year RFS and OS rates highlight the potential efficacy of this treatment strategy.

## 5 Conclusion

Our study demonstrates promising results with acceptable safety and activity of perioperative avelumab with nCRT in locally advanced resectable E/GEJ cancers. It adds to a growing body of literature of utilizing immunotherapy in management of E/GEJ cancers. While our study provides valuable insights, the complexities of treatment sequencing and ongoing trials underscore the need for continued research to refine the management of locally advanced esophageal cancer.

## Author Contributions

**Nataliya V. Uboha:** conceptualization, data curation, formal analysis, investigation, writing–original draft. **Mustafa M. Basree:** data curation, writing–original draft. **Jens C. Eickhoff:** data curation, formal analysis, writing–review and editing. **Dustin A. Deming:** methodology, formal analysis, writing–review and editing. **Kristina Matkowskyj:** Data curation, formal analysis, writing–review and editing. **James Maloney:** investigation, writing–review and editing. **Daniel McCarthy:** investigation, writing–review and editing. **Malcolm DeCamp:** investigation, writing–review and editing. **Noelle LoConte:** investigation, writing–review and editing. **Philip B. Emmerich:** data curation, formal analysis. **Sean Kraus:** data curation, formal analysis. **Monica A. Patel:** writing–review and editing. **Jeremy D. Kratz:** conceptualization, writing–review and editing. **Sam J. Lubner:** conceptualization, writing–review and editing. **Newton Hurst:** writing—review and editing, supervision. **Michael F. Bassetti:** conceptualization, data curation, methodology, investigation, writing–review and editing, supervision.

## Synopsis

This is a phase 2 trial evaluating the addition of avelumab to standard treatment of E/GEJ cancers. Avelumab in combination with chemoradiation did not result in unexpected toxicities. Addition of perioperative avelumab resulted in promising activity against resectable E/GEJ cancers.

## Supporting information

Supporting information.

## Data Availability

The data that support the findings of this study are available from the corresponding author upon reasonable request.
