# Supplementary material for: Phase I/II Trial of Perioperative Avelumab in Combination With Chemoradiation in the Treatment of Stage II/III Resectable Esophageal and Gastroesophageal Junction Cancer
Source: J Surg Oncol. 2025 Jan 5;131(7):1293–301. doi: 10.1002/jso.28070 (PMC12186108; doi:10.1002/jso.28070)
Supplement: Supplementary file 1 — Supporting information. [file JSO-131-1293-s001.docx]

Supplementary Table 1.

*Correlations between stage, complete path response, recurrence, regression score vs. VCAN, Vkine, VPP, and CD8*

|  |  | Correlation Coefficient^1^ | p-value |  |
| --- | --- | --- | --- | --- |
| Stage | VCAN | 0.06 | 0.805 |  |
|  | Vkine | -0.13 | 0.574 |  |
|  | VPP | -0.19 | 0.436 |  |
|  | CD8 | -0.01 | 0.963 |  |
| Complete Path Response | VCAN | -0.25 | 0.256 |  |
|  | Vkine | -0.05 | 0.838 |  |
|  | VPP | 0.19 | 0.436 |  |
|  | CD8 | -0.35 | 0.090 |  |
| Recurrence | VCAN | -0.39 | 0.083 |  |
|  | Vkine | -0.22 | 0.354 |  |
|  | VPP | 0.04 | 0.883 |  |
|  | CD8 | -0.11 | 0.594 |  |
|  |  |  |  |  |
| Regression Score |  | Correlation Coefficient^2^ | p-value |  |
|  | VCAN | 0.30 | 0.222 |  |
|  | Vkine | 0.01 | 0.960 |  |
|  | VPP | -0.29 | 0.244 |  |
|  | CD8 | 0.45 | 0.064 |  |

^1^:Kenall Tau correlation

^2^:Spearman’s rank correlation
